# A unique hybrid characteristic having both pro- and anti-inflammatory phenotype transformed by repetitive low-dose lipopolysaccharide in C8-B4 microglia

**DOI:** 10.1038/s41598-020-65998-8

**Published:** 2020-06-02

**Authors:** Haruka Mizobuchi, Kazushi Yamamoto, Shoko Tsutsui, Masafumi Yamashita, Yoko Nakata, Hiroyuki Inagawa, Chie Kohchi, Gen-Ichiro Soma

**Affiliations:** 1 Control of Innate Immunity, Technology Research Association, Kagawa, Japan; 2 Macrophi Inc., Kagawa, Japan; 3 Research Institute for Healthy Living, Niigata University of Pharmacy and Applied Life Sciences, Niigata, Japan

**Keywords:** Cell biology, Immunology

## Abstract

Although lipopolysaccharide (LPS) is regarded as an inducer of inflammation, previous studies have suggested that repetitive low-dose LPS has neuroprotective effects via immunomodulation of microglia, resident macrophages of brain. However, microglia transformed by the stimulus of repetitive low-dose LPS (REPELL-microglia) are not well characterized, whereas microglia transformed by repetitive high-dose LPS are well studied as an endotoxin tolerance model in which the induction of pro-inflammatory molecules is suppressed. In this study, to characterize REPELL-microglia, the gene expression and phagocytic activity of REPELL-microglia were analyzed with the murine C8-B4 microglia cell line. The REPELL-microglia were characterized by a high expression of pro-inflammatory molecules (*Nos2*, *Ccl1*, IL-12B, and CD86), anti-inflammatory molecules (IL-10, *Arg1*, *Il13ra2*, and Mrc1), and neuroprotective molecules (*Ntf5*, *Ccl7*, and *Gipr*). In addition, the phagocytic activity of REPELL-microglia was promoted as high as that of microglia transformed by single low-dose LPS. These results suggest the potential of REPELL-microglia for inflammatory regulation, neuroprotection, and phagocytic clearance. Moreover, this study revealed that gene expression of REPELL-microglia was distinct from that of microglia transformed by repetitive high-dose LPS treatment, suggesting the diversity of microglia transformation by different doses of LPS.

## Introduction

Lipopolysaccharide (LPS) is a glycolipid expressed in the outer membrane of gram-negative bacteria. Because LPS is an inducer of potent systemic inflammation when experimentally administered intravenously or intraperitoneally, LPS has been known as an endotoxin since its discovery. By contrast, we have demonstrated the beneficial effects of orally administered LPS to improve pathological conditions in various pathogenesis models^1–3^. Our results have shown that transmucosal administration of LPS at an appropriate concentration, frequency, and exposure route does not contribute to the exacerbation of inflammation but, instead, to the maintenance of homeostasis.

Recently, we reported that Alzheimer’s disease-related cognitive impairment was improved by oral administration of LPS (1 mg/kg/day) in mice, and that suppression of the pro-inflammatory cytokine interleukin (IL)-6 together with promotion of anti-inflammatory cytokine IL-10 were induced by orally administered LPS^4^. In addition, previous reports have shown that microglia, the resident macrophages (MΦ) of brain, contribute to amyloid β clearance by promoting phagocytosis^5,6^. These results suggest that microglia are transformed by oral LPS treatment and gain the neuroprotective characteristics of high phagocytic activity and inflammatory regulation. In this regard, other groups have reported that neuropathology was improved by intraperitoneal administration of repetitive low-dose LPS^7–10^. Contrary to the detrimental effect of intraperitoneal administration of a single, high-dose of LPS to exacerbate Aβ accumulation, neuronal damage, and cognitive impairment^7,11,12^, they showed that the opposing effects on the neuropathy by LPS (that is, worsening effect by single LPS treatment and healing effect by repetitive LPS treatment) were associated with different microglial characteristics. Considering these results, it is reasonable to suppose that one of the requirements to induce a neuroprotective effect by LPS is repetitive and low-dose administration, and that the microglia transformed under this LPS condition play a critical role in the maintenance of brain homeostasis. Thus, characterization of microglia transformed by the stimulus of repetitive low-dose LPS (REPELL-microglia) provides us with important clues to a breakthrough for elucidating the unclear mechanism of homeostasis maintenance by oral administration of LPS.

However, little is known about the detailed profiles of REPELL-microglia because most studies on the characterization of LPS-stimulated microglia have been performed with a model of severe inflammation induced by high-dose LPS. The purpose of this study is to characterize murine REPELL-microglia. First, we characterized REPELL-microglia by phagocytic activity and their expression of pro-inflammatory molecules, anti-inflammatory molecules, and neuroprotective molecules. Then, to verify characteristics of REPELL-microglia that are unique to low-dose LPS treatment, REPELL-microglia were compared with microglia transformed by repetitive high-dose LPS. Results demonstrated that REPELL-microglia are characterized by high phagocytic activity and high expression of pro-inflammatory, anti-inflammatory, and neuroprotective molecules, which are unique to low-dose LPS treatment.

## Results

### Pro-inflammatory molecules *Nos2*, *Ccl1*, IL-12B, and CD86 are highly expressed in REPELL-microglia

**Figure 1:**
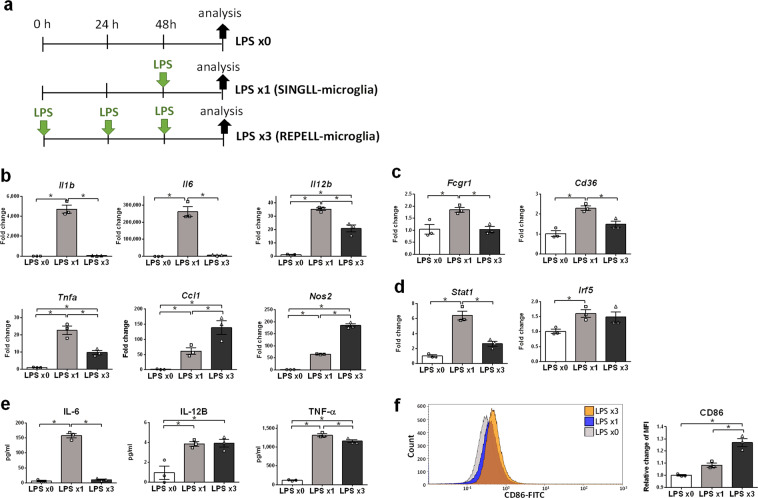
Pro-inflammatory molecules *Nos2*, *Ccl1*, IL-12B, and CD86 are highly expressed in REPELL-microglia. (**a**) The experimental schedule of LPS treatment. C8-B4 microglia cells were treated with low-dose LPS (1 ng/mL) one or three times (n = 3, in triplicate). (**b,c**) relative mRNA expression of pro-inflammatory molecules was measured by real-time RT-PCR using the 2^−∆∆Ct^ method 4 h after the last LPS treatment. Data were normalized to GAPDH and expressed as a relative fold change over untreated cells. (**b**) Cytokines and metabolic enzymes, (**c**) cell surface receptors, and (**d**) transcriptional regulators. (**e**) The secretion of IL-12B was promoted by repetitive low-dose LPS in microglia. Cytokine levels of TNF-α, IL-12B, and IL-6 in culture supernatant were measured by ELISA 30 h after the last LPS treatment. (**f**) CD86 expression on cell surfaces was promoted by repetitive low-dose LPS in microglia. The cell surface antigen CD86 was stained, and the MFI of 50,000 counted cells was assessed 6 h after the last LPS treatment using a Beckman Coulter Gallios flow cytometer and Kaluza software. Data are expressed as the relative fold change over untreated cells. Mean ± SE of each group are shown. Data are representative of three independent experiments. LPS x0, no treatment; LPS x1, single treatment with low-dose LPS; LPS x3, treatment with low-dose LPS three times every 24 h. *p < 0.05 for one-way ANOVA with Tukey’s post-hoc correction for multiple comparisons.

Abbreviations of genes and molecules used for characterization of REPELL-microglia in this study are listed in Supplementary Table 1. C8-B4 microglia is the most widely used rodent microglia cell line and similarities in characteristics between the C8-B4 cell line and primary microglia have been reported^13–15^. To characterize REPELL-microglia, C8-B4 microglia were treated with low-dose LPS (1 ng/mL) one or three times (n = 3, in triplicate) (Fig. 1a), and the expression of pro-inflammatory molecules in REPELL-microglia was compared to that in microglia transformed by a stimulus of single low-dose LPS (SINGLL-microglia). Quantitative RT-PCR revealed that SINGLL-microglia upregulated the expression of pro-inflammatory genes such as cytokines (*Il1b*, *Il6*, *Il12b*, *Tnfa*, and *Ccl1*), arginine-metabolic enzyme *Nos2* (Fig. 1b), cell surface receptors (*Fcgr1* and *Cd36*) (Fig. 1c), and transcriptional regulators (*Stat1* and *Irf5*) (Fig. 1d). Most pro-inflammatory gene expressions in REPELL-microglia were not comparable to those of SINGLL-microglia. However, exceptionally, expression of arginine-metabolic enzyme *Nos2* and chemokine *Ccl1* was significantly more upregulated in REPELL-microglia than in SINGLL-microglia. Next, the protein levels of representative cytokines in culture supernatant were measured (Fig. 1e). Consistent with mRNA expression for these groups, the secretion of IL-6 and TNF-α was promoted in SINGLL-microglia and suppressed in REPELL-microglia. However, the secretion of IL-12B was promoted in REPELL-microglia at the same intensity as that of SINGLL-microglia. Besides this, the protein expression of CD86 on the cell surfaces of REPELL-microglia was higher than that of the untreated controls and SINGLL-microglia (Fig. 1f). These results demonstrated that REPELL-microglia is characterized by high expression of *Nos2*, *Ccl1*, IL-12B, and CD86.

### Anti-inflammatory molecules IL-10, *Arg1*, *Il13ra2*, and Mrc1 are highly expressed in REPELL-microglia

**Figure 2:**
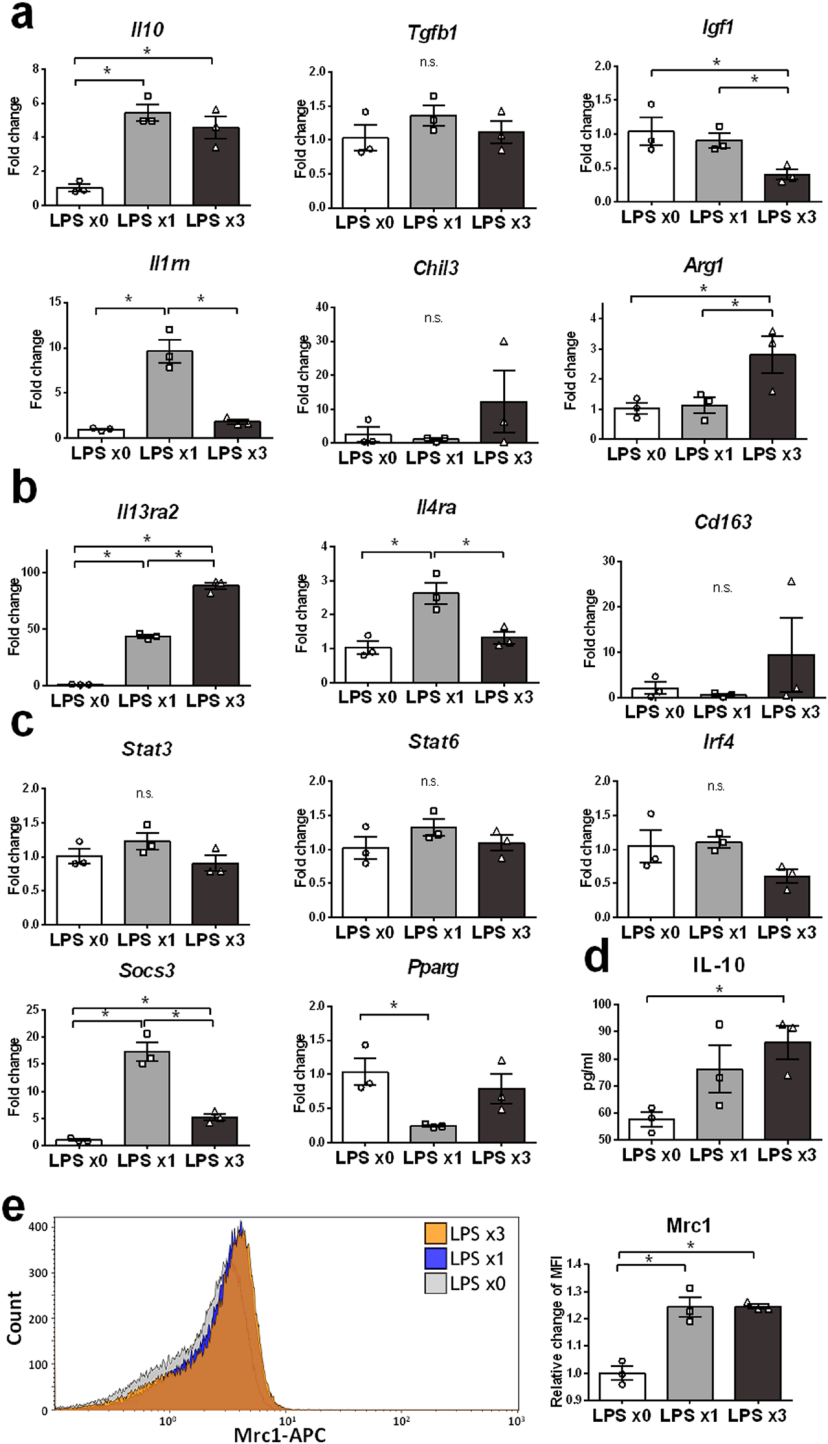
Anti-inflammatory molecules IL-10, *Arg1*, *Il13ra2*, and Mrc1 are highly expressed in REPELL-microglia. (**a**–**c**) C8-B4 microglia were treated with low-dose LPS (1 ng/mL) one or three times (n = 3, in triplicate), and relative mRNA expression of anti-inflammatory molecules was measured by real-time RT-PCR using the 2^−∆∆Ct^ method 4 h after the last LPS treatment. Data were normalized to GAPDH and expressed as the relative fold change over untreated cells. (**a**) Cytokines and metabolic enzymes, (**b**) cell surface receptors, and (**c**) transcriptional regulators. (**d**) Secretion of IL-10 was promoted by repetitive low-dose LPS in microglia. The levels of IL-10 in culture supernatant were measured by ELISA 30 h after the last LPS treatment. (**e**) Mrc1 expression on cell surfaces was promoted by repetitive low-dose LPS in microglia. The cell surface antigen Mrc1 was stained, and the mean fluorescence intensity (MFI) of 50,000 counted cells was assessed 6 h after the last LPS treatment using a Beckman Coulter Gallios flow cytometer and Kaluza software. Data are expressed as the relative fold change over untreated cells. Mean ± SE of each group are shown. Data are representative of three independent experiments. LPS x0, no treatment; LPS x1, single treatment with low-dose LPS; LPS x3, treatment with low-dose LPS three times every 24 h. *p < 0.05 for one-way ANOVA with Tukey’s post-hoc correction for multiple comparisons. n.s., not significant.

Expression of anti-inflammatory molecules in REPELL-microglia was analyzed. Quantitative RT-PCR showed that *Il10, Arg1*, and *Il13ra2* were highly expressed in REPELL-microglia (Fig. 2a–c). Gene expression of the anti-inflammatory cytokine *Il10* was not suppressed in REPELL-microglia and maintained the same level of promotion as that found in SINGLL-microglia (Fig. 2a). Expressions of arginine-metabolic enzyme *Arg1* and cell surface receptor *Il13ra2* were significantly upregulated in REPELL-microglia than in SINGLL-microglia (Fig. 2a,b). By contrast, REPELL-microglia did not upregulate expression of the other anti-inflammatory molecules such as anti-inflammatory cytokines (*Tgfb*, *Igf1*, *Il1rn*, and *Chil3*) (Fig. 2a), cell surface receptors (*Il4ra* and *Cd163*) (Fig. 2b), and transcriptional regulators (*Stat3*, *Stat6*, *Irf4*, *Socs3*, and *Pparg*) (Fig. 2c). In addition, REPELL-microglia promoted the secretion of IL-10 (Fig. 2d) into the culture medium compared with the untreated controls. Protein expression of Mrc1 (Fig. 2e) on the cell surface of REPELL-microglia was higher than that of the untreated controls and was comparable to that of SINGLL-microglia. Together, these results showed that REPELL-microglia highly express IL-10, *Arg1*, *Il13ra2*, and Mrc1.

### Neuroprotective molecules *Ntf5*, *Gipr*, and *Ccl7* are highly expressed in REPELL-microglia

**Figure 3:**
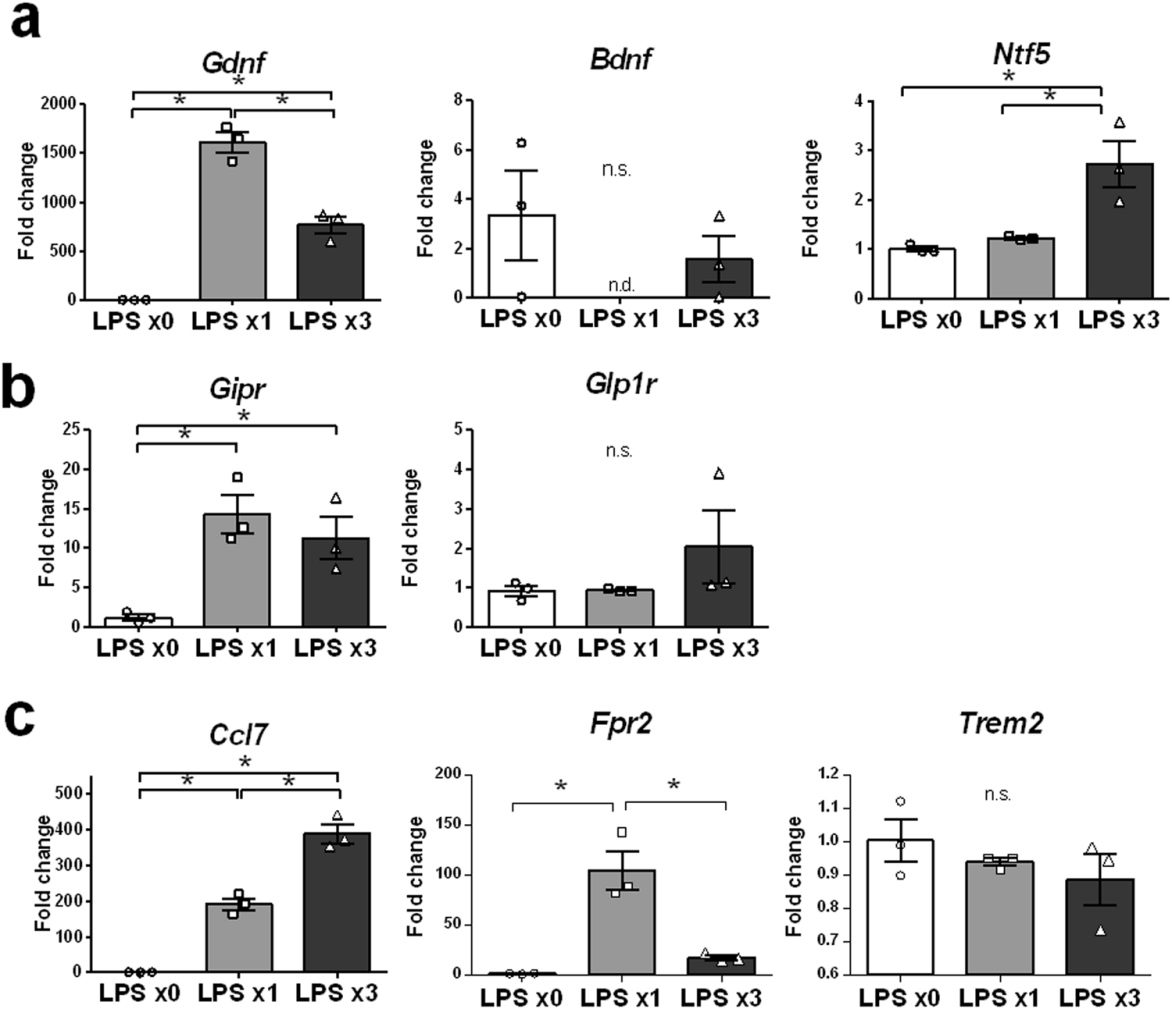
Neuroprotective molecules such as *Ntf5*, *Gipr*, and *Ccl7* highly expressed in REPELL-microglia. C8-B4 microglia were treated with low-dose LPS (1 ng/mL) one or three times (n = 3, in triplicate), and relative mRNA expression of neuroprotective genes was measured by real-time RT-PCR using the 2^−∆∆Ct^ method 4 h after the last LPS treatment. Data were normalized to GAPDH and expressed as the relative fold change over untreated cells. (**a**) Neurotrophic molecules, (**b**) incretin receptors, and (**c**) cell surface receptors associated with neuroprotection. Mean ± SE of each group are shown. Data are representative of three independent experiments. LPS x0, no treatment; LPS x1, single treatment with low-dose LPS; LPS x3, treatment with low-dose LPS three times every 24 h. *p < 0.05 for one-way ANOVA with Tukey’s post-hoc correction for multiple comparisons. n.s., not significant.

For further characterization of REPELL-microglia, we analyzed expression of neuroprotective genes was analyzed such as neurotrophic molecules (*Gdnf, Bdnf*, and *Ntf5*) (Fig. 3a), incretin receptors (*Gipr* and *Glp1r*) (Fig. 3b), and cell surface receptors associated with neuroprotection (*Ccl7, Fpr2*, and *Trem2*) (Fig. 3c). Expression of neurotrophin 5 (*Ntf5*) and C-C motif chemokine ligand 7 (*Ccl7*) was upregulated in REPELL-microglia compared with the untreated controls and SINGLL-microglia (Fig. 3a,c). Expression of gastric inhibitory polypeptide receptor (*Gipr*) was upregulated in REPELL-microglia to the same high levels as those of SINGLL-microglia (Fig. 3b). Expression of the other neuroprotective genes investigated in this study was suppressed or not changed in REPELL-microglia. This is the first known identification of that *Ntf5, Gipr*, and *Ccl7* being highly expressed in REPELL-microglia.

### Maintaining promotion of phagocytic activity in REPELL-microglia

**Figure 4:**
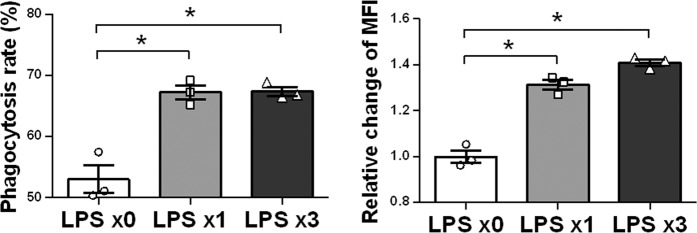
Maintaining promotion of phagocytic activity in REPELL-microglia. C8-B4 microglia were treated with low-dose LPS (1 ng/mL) one or three times (n = 3, in triplicate). At 24 h after the last LPS treatment, cells were incubated with fluorescent latex beads for 3 h at a cell:bead ratio of 1:5. The phagocytosis rate (left panel) and mean fluorescence intensity (MFI) (right panel) were assessed using a Beckman Coulter Gallios flow cytometer with Kaluza software. The MFI of cells phagocytizing beads was expressed as the relative fold change over untreated cells. Mean ± SE of each group are shown. Data are representative of three independent experiments. LPS x0, no treatment; LPS x1, single treatment with low-dose LPS; LPS x3, treatment with low-dose LPS three times every 24 h. *p < 0.05 for one-way ANOVA with Tukey’s post-hoc correction for multiple comparisons.

Phagocytosis is one of the pivotal functions of microglia to remove xenobiotics and waste products, maintaining brain homeostasis. To assess phagocytic activity, REPELL-microglia were incubated with fluorescent latex beads, and the phagocytosis rate and mean fluorescence intensity (MFI) of phagocytosed beads in the cells were measured (Fig. 4). Compared with untreated controls (53.0% ± 1.9%), the phagocytosis rate of REPELL-microglia (67.4% ± 0.9%) was promoted to that of SINGLL-microglia (67.2% ± 0.6%). The MFI of phagocytosed beads in the cells also showed same tendency. The MFI of REPELL-microglia was 1.4 times higher than the untreated controls, and was comparable to the MFI of SINGLL-microglia. These results therefore demonstrated that REPELL-microglia have high phagocytic activity against foreign substances.

### The gene expression pattern of REPELL-microglia is unique to the low-dose LPS condition

**Figure 5:**
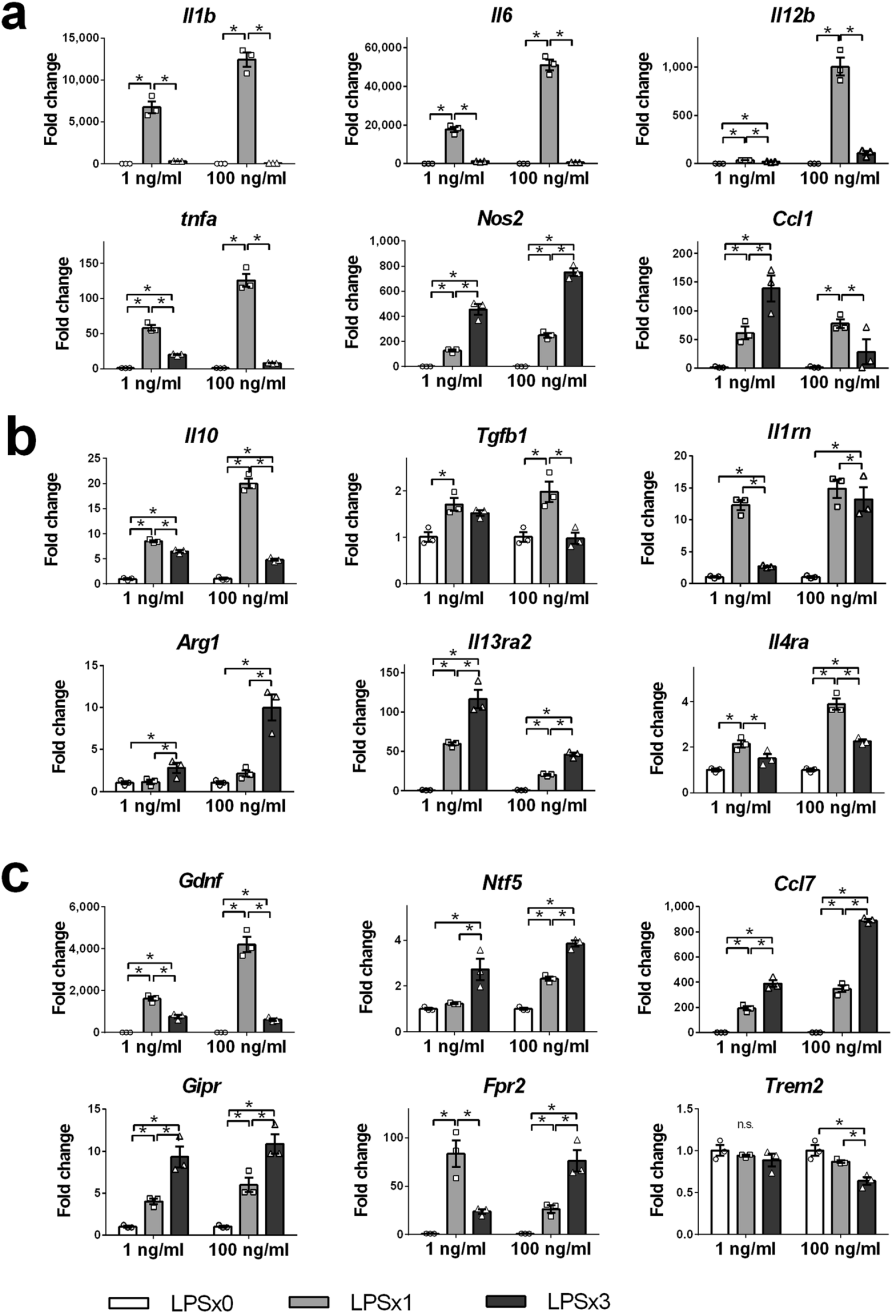
Distinct gene expression induced by low-dose and high-dose LPS characterized by *Ccl1*, *Il1rn*, *Fpr2*, and *Trem2* expression in microglia. C8-B4 microglia were treated with low- or high-dose LPS (1 or 100 ng/mL) one or three times (n = 3, in triplicate), and relative mRNA expression at 4 h after the last LPS treatment was measured by real-time RT-PCR using the 2^−∆∆Ct^ method. Data were normalized to GAPDH and expressed as the relative fold change over untreated cells. (**a**) pro-inflammatory molecules, (**b**) anti-inflammatory molecules, and (**c**) neuroprotective genes. Mean ± SE of each group are shown. Data are representative of three independent experiments. LPS x0, no treatment; LPS x1, single treatment with low-dose LPS; LPS x3, treatment with low-dose LPS three times every 24 h. *p < 0.05 for one-way ANOVA with Tukey’s post-hoc correction for multiple comparisons. n.s., not significant.

To test whether the heretofore mentioned characteristics of REPELL-microglia were unique to low-dose LPS treatment, the mRNA expression of REPELL-microglia (1 ng/mL LPS) was compared with that of microglia transformed by repetitive high-dose LPS (100 ng/mL LPS) (Fig. 5). Gene expression patterns of *Ccl1*, *Il1rn*, *Fpr2*, and *Trem2* were different between the two groups. The level of *Ccl1* was upregulated in REPELL-microglia but suppressed during repetitive high-dose LPS treatment (Fig. 5a). The level of *Il1rn* was suppressed in REPELL-microglia compared with that of SINGLL-microglia, whereas it was promoted by repetitive high-dose LPS to as high as the single high-dose LPS group (Fig. 5b). The level of *Fpr2* was suppressed in REPELL-microglia compared with SINGLL-microglia and in contrast to its upregulation by repetitive high-dose LPS. *Trem2* level was not changed in REPELL-microglia, but it was downregulated by repetitive high-dose LPS (Fig. 5c). Expression of the other genes showed the same tendency between low-dose and high-dose LPS treatments. Thus, gene expression induced by repetitive LPS differed depending on LPS concentration, indicating that the gene expression pattern of REPELL-microglia is unique to its low-dose LPS treatment.

## Discussion

Previous studies suggest that administration of repetitive low-dose LPS contributes to neuroprotection by inducing microglia with neuroprotective phenotype^7–10,16^. However, few studies have focused on the characterization of REPELL-microglia. In the present study, we characterized REPELL-microglia by high expression of pro-inflammatory molecules (*Nos2*, *Ccl1*, IL-12B, and CD86: Fig. 1), anti-inflammatory molecules (IL-10, *Arg1*, *Il13ra2*, and Mrc1: Fig. 2), and neuroprotective molecules (*Ntf5*, *Ccl7*, and *Gipr*: Fig. 3). For these representative molecules such as IL-6, TNF-α, IL-12B, and IL-10, protein expression levels (IL-6, TNF-α, IL-12B: Fig. 1e, and IL-10: Fig. 2d) were confirmed to be almost correlative with mRNA expression (*Il6*, *Tnfa*, *Il12b*: Fig. 1b, and *Il10*: Fig. 2a). It is generally known that a single LPS treatment induces pro-inflammatory molecules, while repetitive LPS treatment suppresses the induction of pro-inflammatory molecules, a phenomenon known as “LPS tolerance”. Indeed, it has been reported that pro-inflammatory molecules such as IL-1β, IL-6, and TNF-α are suppressed in microglia by repetitive high-dose LPS treatment^17–22^. Although IL-1β, IL-6, and TNF-α were suppressed in REPELL-microglia similarly to data of past reports with a high-dose LPS model, REPELL-microglia did not exhibit global downregulation of pro-inflammatory molecules, but instead showed high expression of *Nos2*, *Ccl1*, IL-12B, and CD86 (Fig. 1). The mRNA expression of IL-12B was slightly suppressed in REPELL-microglia, whereas the protein level was as high as that of SINGLL-microglia, and this may be associated with a protein stabilization mechanism at the posttranscriptional level^23–25^. REPELL-microglia expression of anti-inflammatory molecules was partially consistent with previous reports of upregulated IL-10, Arg1, and Mrc1 by repetitive high-dose LPS^8,26,27^. The co-expression of pro-inflammatory and anti-inflammatory molecules is one of the unique characteristics of REPELL-microglia. Because the combination of autologous inactivated tumor cells expressing IL-12 and IL-10 was reported to induce synergic tumor remission by controlling local inflammation^28^, it is suggested that REPELL-microglia expressing both IL-12 and IL-10 have the potential for regulating inflammation. In addition, REPELL-microglia highly express neuroprotective molecules *Ntf5*, *Gipr*, and *Ccl7* (Fig. 3). NTF5^29^ and GIPR^30–32^ have neuroprotective effects via their anti-oxidant and anti-apoptosis qualities during encephalitis, and CCL7 associated with neuron differentiation^33^, suggesting the neuroprotective potential of REPELL-microglia through these factors. It has been reported that microglia “memorize” repetitive LPS stimulation and transform into neuroprotective cells^7^, and the neuroprotective molecules identified in this study may be involved in this mechanism. Meanwhile, microRNA (miRNA) is one of the mechanisms by which synthesis of protein from mRNA is suppressed. It has been reported that miRNA-146a, miRNA-155, miRNA-221, miRNA-125b, miRNA-132, miRNA-579, and miRNA-21 are involved in LPS tolerance^34–36^. In this context, it was confirmed by miRNA database (microRNA.org) that molecules with high mRNA expression in REPELL-microglia such as *Nos2, Ccl1* (Fig. 1b), *Arg1, Il13ra2* (Fig. 2a,b), *Ntf5, Ccl7*, and *Gipr* (Fig. 3a–c) were not targets of the miRNAs induced during LPS tolerances as described above. Therefore, it is suggested that translation of mRNA of *Nos2, Ccl1, Arg1, Il13ra2, Ntf5, Ccl7*, and *Gipr* into protein is not likely to be suppressed under the conditions of our study at least by this miRNA mechanism.

REPELL-microglia furthermore showed high phagocytic activity (Fig. 4). It was reported that microglia improved Alzheimer’s disease-related pathologies by promoting the phagocytosis of Aβ^7^, suggesting that REPELL-microglia with high phagocytic activity contributes to the clearance of brain xenobiotics that cause neuropathy. TREM2 is one of the molecules involved in promotion of phagocytosis and suppression of pro-inflammatory molecules such as TNF-α and NOS2 in microglia^37,38^. In REPELL-microglia, however, the expression of *Trem2* was unchanged, whereas *Nos2* expression and phagocytosis were promoted. Therefore, it is suggested that phagocytosis of REPELL-microglia is promoted by a TREM2-independent mechanism. Indeed, phagocytosis is a remarkably complex process involving various phagocytic receptors and actin remodeling^39^. Because IL-12B and TNF-α promote NOS2 induction^40,41^, and NOS2 has a pivotal role not only in intracellular killing but also in phagocytic activity^42^, it is suggested that these molecules are associated with high phagocytic activity of REPELL-microglia.

Taken together, the present study has demonstrated for the first time that REPELL-microglia are characterized by high phagocytic activity and high expression of pro-inflammatory, anti-inflammatory, and neuroprotective molecules. Interestingly, the unique characteristics of REPELL-microglia are suggested to contribute to maintaining brain homeostasis, almost as if they are “repelling disease” both in name and reality. In addition, it was reported that mild oxygen-glucose deprivation induced microglia to show an anti-inflammatory and protective phenotype *in vitro*^43^, which is a phenomenon similar to that of REPELL-microglia in our study. Therefore, these data suggest that microglial protective phenotype can be induced by “mild” stimulation whether LPS or oxygen-glucose deprivation.

Importantly, the characteristics of REPELL-microglia are consistent with those of microglia induced by orally administered LPS in an Alzheimer’s disease mouse model *in vivo*^16^ in terms of high phagocytic activity and high IL-10 expression. In this context, another study has reported that microglia transformed by intraperitoneal administration of repetitive low-dose LPS also exhibit the characteristics of enhanced Aβ phagocytosis and high IL-10 expression, again consistent with the characteristics of REPELL-microglia^7^. Therefore, characteristics of REPELL-microglia as described above may partially reflect those of microglia transformed by oral administration of LPS *in vivo*. Because it was reported that intraperitoneal administration of IL-10 can induce microglia to express high levels of IL-10 as in the case of intraperitoneal administration of repetitive low-dose LPS^7^, IL-10 also may be a key mediator common to the induction mechanisms of both REPELL-microglia *in vitro* and microglia transformed by oral administration of LPS *in vivo*.

Moreover, the present study demonstrates that REPELL-microglia are not simply a miniature version of microglia transformed by repetitive high-dose LPS^10^ but exhibit fundamentally distinct characteristics (Fig. 5). The results suggest that the condition of low-dose LPS is an important molecule to induce transformation into REPELL-microglia. Microglia play an important role in innate immunity for homeostatic maintenance by diverse transformation adapted to various microenvironment conditions^44–46^, and it has been recently reported that the diversity of microglia can be transformed with different ligands, diseases, and time courses^45–49^. However, little has been recognized about the diversity of microglia transformation by different doses of LPS, and the phenotype of microglia transformed by low-dose LPS often has been considered to be simply a reduced version of that of high-dose LPS^10^. Therefore, the present study provides a new perspective of the diversity of microglia transformation by different doses of LPS. This concept is also supported by a recent report that pre-treatment with low-dose LPS (1 fg/mL) primes microglia to enhance pro-inflammatory cytokine production in response to subsequent stimulation with high-dose LPS (100 ng/mL) mediated by PI3Kγ^50^. In addition, other report showed in macrophages that pre-stimulation of low-dose LPS (5 pg/mL) induces RelB degradation in contrast to RelB induction that occurs during pre-stimulation of high-dose LPS (100 ng/mL)^51^, suggesting that different intracellular signal transductions operate in microglia stimulated with low dose versus high-dose LPS.

**Figure 6:**
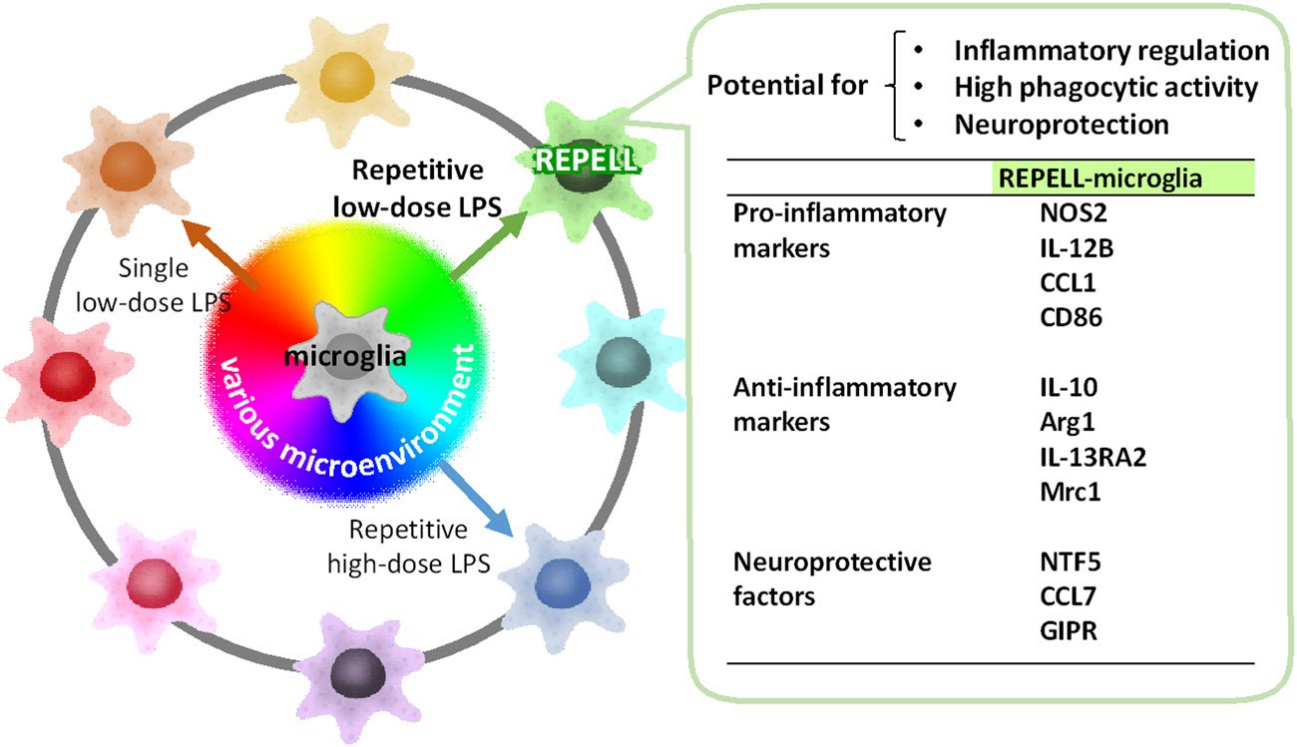
Model of a unique hybrid characteristic having both pro- and anti-inflammatory phenotype transformed by repetitive low-dose lipopolysaccharide in C8-B4 microglia. REPELL-microglia is characterized by high phagocytic activity and mixed expression of pro-inflammatory molecules (NOS2, IL-12B, CCL1, and CD86), anti-inflammatory molecules (IL-10, Arg1, IL-13RA2, and Mrc1), and neuroprotective molecules (NTF5, CCL7, and GIPR). Gene expression patterns of REPELL-microglia are distinct from that of microglia transformed by repetitive high-dose LPS, indicating the diverse transformation of microglia by different LPS concentrations.

In conclusion, the present study demonstrates that REPELL-microglia are uniquely characterized by high phagocytic activity and high expression of pro-inflammatory, anti-inflammatory, and neuroprotective molecules (Fig. 6), suggesting the potential of REPELL-microglia to “repel disease” and to promote the maintenance of brain homeostasis. Moreover, this study revealed that low-dose LPS is a key factor for inducing REPELL-microglia, indicating the diversity of microglia transformation by different doses of LPS. Appropriate LPS treatment with repetitive low-dose administration may be expected to become a new therapeutic or preventive target for repelling various neurological disorders by priming the teleological transformation of microglia.

## Methods

### Cell culture and LPS treatment

The murine microglial cell line C8-B4 was purchased from the American Type Culture Collection (Manassas, VA, USA). A minimum sample size for one-way ANOVA was determined using power analysis in a prior examination with the statistical analysis software R (R Core Team (2019). R: A language and environment for statistical computing. R Foundation for Statistical Computing, Vienna, Austria.). C8-B4 microglia (2 × 10^5^ cells/mL) were seeded in 12-well tissue culture plates (n = 3, in triplicate) and cultured in Dulbecco’s modified Eagle’s medium (Wako, Osaka, Japan) supplemented with 10% fetal bovine serum (Sigma-Aldrich, St Louis, MO, USA), 100 U/mL penicillin and 100 μg/mL streptomycin (Life Technologies, Carlsbad, CA, USA), at 37 °C in 5% CO_2_. C8-B4 microglia were treated with or without LPS (1 or 100 ng/mL, purified LPS derived from *Pantoea agglomerans*, Macrophi Inc., Kagawa, Japan) (Fig. 1a). The concentration of LPS was set as low as 1 ng/mL because previous *in vivo* studies suggest that serum LPS levels were estimated to be at most 1 ng/mL after oral administration of LPS at a concentration of 1 mg/kg/day^52^. For repetitive treatment with LPS, cells received fresh medium containing LPS three times every 24 h. For single treatment with LPS, cells received fresh medium without LPS for the first 48 h and then received a one-time dose of LPS in fresh medium. Samples were collected at the indicated time points.

### Quantitative RT-PCR

Four hours after the last LPS treatment, RNA was extracted from cells by RNeasy mini kit (QIAGEN, Hilden, Germany) and cDNA was synthesized by reverse transcription using ReverTra Ace qPCR RT Master Mix (TOYOBO, Osaka, Japan) according to the manufacturers’ instructions. Real-time PCR assay was carried out using 2 μL of cDNA as the template and 10 μL of Power SYBR Green PCR Master Mix (Thermo Fisher Scientific, Waltham, MA, USA) on the Stratagene Mx 3005 P QPCR System (Agilent Technologies, Santa Clara, CA, USA). Primers used in this study are listed in Supplementary Table 2. Data were analyzed by the 2^−∆∆Ct^ method and normalized to GADPH. The thermal cycling conditions for PCR were 95 °C for 10 min for polymerase activation, followed by 45 cycles of 95 °C for 15 sec for denaturation and 60 °C for 1 min for extension.

### Quantification of cytokines by ELISA

Thirty hours after the last LPS treatment, culture supernatants were collected. IL-10, IL-12B, TNF-α, and IL-6 levels in the culture supernatants were measured using commercial sandwich ELISA kits (BioLegend, San Diego, CA, USA) per the manufacturers’ instructions. Absorbance was measured at 450 nm by iMark microplate reader (BIO RAD, Hercules, CA, USA).

### Flow-cytometric analysis of cell surface antigens

Six hours after the last LPS treatment, expressions of cell surface antigens CD86 and Mrc1 were analyzed by flow cytometry as previously described^5^ with minor modifications. In brief, cells were detached by 0.25% trypsin treatment (Life Technologies) and resuspended in PBS containing 0.5% bovine serum albumin and 2 mM EDTA. The cells were stained with FITC-labeled anti-CD86 antibody (eBioscience, San Diego, CA, USA) or APC-labeled anti-Mrc1 antibody (BioLegend) for 30 min at 4 °C. The MFI was measured in a total of 50,000-counted cells using a Beckman Coulter Gallios flow cytometer and Kaluza for Gallios software version 1.3 (Beckman Coulter, Indianapolis, IN, USA).

### Phagocytosis assay

Phagocytic activity was measured by flow cytometry as previously described^5^ with minor modifications. In brief, 24 h after the last LPS treatment, cells were incubated with fluorescent latex beads (Fluoresbrite YG Microspheres 2.0 μm; Polysciences, Warrington, PA, USA) at a cell:bead ratio of 1:5 for 3 h. Cells were washed with PBS to remove non-internalized particles, and detached by 0.25% trypsin treatment (Life Technologies). Cells were resuspended in PBS, and the phagocytosis rate and MFI of phagocytosed beads in the cells were measured using a Beckman Coulter Gallios flow cytometer with Kaluza software (Beckman Coulter).

### Statistical analysis

Statistical analysis was performed using statistical analysis software R and the GraphPad Prism 6.0 software package (GraphPad Software Inc., San Diego, CA, USA). Results were presented as mean ± standard error (SE) of the mean. Differences between the groups were analyzed by one-way ANOVA followed by Tukey’s multiple comparisons test, and a *p* value <0.05 was considered significantly different. All experiments were conducted with at least three independent biological replicates.

## Supplementary information

Supplementary information.
