# Supplementary material for: A unique hybrid characteristic having both pro- and anti-inflammatory phenotype transformed by repetitive low-dose lipopolysaccharide in C8-B4 microglia
Source: Sci Rep. 2020 Jun 2;10:8945. doi: 10.1038/s41598-020-65998-8 (PMC7265460; doi:10.1038/s41598-020-65998-8)
Supplement: Supplementary file 1 — Supplementary information. [file 41598_2020_65998_MOESM1_ESM.pdf]

Supplementary information

A unique hybrid characteristic having both pro- and anti-inflammatory phenotype transformed by repetitive low-dose lipopolysaccharide in C8-B4 microglia

Haruka Mizobuchi[1], Kazushi Yamamoto[1], Shoko Tsutsui[2], Masafumi Yamashita[1], Yoko Nakata[2], Hiroyuki Inagawa[1,2,3], Chie Kohchi[2] & Gen-Ichiro Soma[1,2,3*]

[1] *Control of Innate Immunity, Technology Research Association, Kagawa, Japan*

[2] *Macrophi Inc., Kagawa, Japan*

[3] *Research Institute for Healthy Living, Niigata University of Pharmacy and Applied Life Sciences, Niigata, Japan*

*Corresponding Author: Gen-Ichiro Soma

Address: 2217-16 Hayashi-cho, Takamatsu-shi, Kagawa 761-0301, Japan

Phone: +81-87-813-9201

Fax: +81-87-813-9203

Email address: mizobuchi@shizenmeneki.org

Supplementary Table 1. Abbreviations

Gene name, Protein name	
Arg1, Arg1	Arginase 1
Bdnf, BDNF	Brain derived neurotrophic factor
Ccl1, CCL1	C-C Motif chemokine ligand 1
Ccl7, CCL7	C-C Motif chemokine ligand 7
Chil3, Chil3	Chitinase-like protein 3/YM1
Fcgr1, FcgR1	Fc receptor, IgG, high affinity I
Fpr2, FPR2	Formyl peptide receptor 2
Gapdh, GAPDH	Glyceraldehyde-3-phosphate dehydrogenase
Gdnf, GDNF	Glial cell derived neurotrophic factor
Gipr, GIPR	Gastric inhibitory polypeptide receptor
Glp1r, GLP1R	Glucagon like peptide 1 receptor
Igf1, IGF-1	Insulin like growth factor 1
Il10, IL-10	Interleukin 10
Il12b, IL-12B	Interleukin 12B
Il13ra2, IL-13RA2	Interleukin 13 receptor subunit alpha 2
Il1b, IL-1β	Interleukin 1 beta
Il1rn, IL-1RA	Interleukin 1 receptor antagonist
Il4ra, IL-4RA	Interleukin 4 receptor, alpha
Il6, IL-6	Interleukin 6
Irf4, IRF4	Interferon regulatory factor 4
Irf5, IRF5	Interferon regulatory factor 5
Mrc1, Mrc1	Mannose receptor c-type 1/CD206
Nos2, Nos2	Nitric oxide synthase 2
Ntf5, NTF5	Neurotrophin 5
Pparg, PPARγ	Peroxisome proliferator activated receptor gamma
Socs3, SOCS3	Suppressor of cytokine signaling 3
Stat1, STAT1	Signal transducer and activator of transcription 1
Stat3, STAT3	Signal transducer and activator of transcription 3
Stat6, STAT6	Signal transducer and activator of transcription 6
Tgfb1, TGF-β	Transforming growth factor beta 1
Tnfa, TNF-α	Tumor necrosis factor alpha
Trem2, TREM2	Triggering receptor expressed on myeloid cells 2

Supplementary Table 2. List of primers used for quantitative RT-PCR.

Gene	Forward	Reverse
Arg1	5′-CTCCAAGCCAAAGTCCTTAGAG-3′	5′-AGGAGCTGTCATTAGGGACATC-3′
Bdnf	5′-GCGCCCATGAAAGAAGTAAA-3′	5′-TCGTCAGACCTCTCGAACCT-3′
Ccl1	5′-GGATGTTGACAGCAAGAGCA-3′	5′-TAGTTGAGGCGCAGCTTTCT-3′
Ccl7	5′-AAGATCCCCAAGAGGAATCTCA-3′	5′-CAGACTTCCATGCCCTTCTTT-3′
Cd163	5′-GGCACTCTTGGTTTGTGGAG-3′	5′-GCCTTTGAATCCATCTCTTGG-3′
Cd36	5′-GCCAAGCTATTGCGACATGA-3′	5′-AAAAGAATCTCAATGTCCGAGACTTT-3′
Chil3	5′-CATGAGCAAGACTTGCGTGAC-3′	5′-GGTCCAAACTTCCATCCTCCA-3′
Fcgr1	5′-GGGAAGACACCGCTACACAT-3′	5′-GGAGATGACA CGGATGCTCT-3′
Fpr2	5′-TCTACCATCTCCAGAGTTCTGTGG-3′	5′-TTACATCTACCACAATGTGAACTA-3′
Gapdh	5′-CGACTTCAACAGCAACTCCCACTCTTCC-3'	5′-TGGGTGGTCCAGGGTTTCTTACTCCTT-3′
Gdnf	5′-TGACTCCAATATGCCTGAAGATTATC-3′	5′-AATGGTGGCTTGAATAAAATCCA-3′
Gipr	5′-CCGCGCTTTTCGTCAT-3′	5′-CCACCAAATGGCTTTGACTT-3′
Glp1r	5′-TCAGAGACGGTGCAGAAATG -3′	5′- CAGCTGACATTCACGAAGGA-3′
Igf1	5′-TCATGTCGTCTTCACACCTCTTCT-3′	5′-CCACACACGAACTGAAGAGCAT-3′
Il10	5′-GCTGGACAACATACTGCTAACC-3′	5′-CCCAAGTAACCCTTAAAGTCCTG-3′
Il12b	5′-ACAGCACCAGCTTCTTCATCAG-3′	5′-TCTTCAAAGGCTTCATCTGCAA-3′
Il13ra2	5′-CGTACGCATTTGTCAGAGCA-3′	5′-AGGTTTCCAAGAGCAGACCA-3′
Il1b	5′-GAAAGACGGCACACCCACCCT-3′	5′-GCTCTGCTTGTGAGGTGCTGATGTA-3′
Il1rn	5′-GACCCTGCAAGATGCAAGCC-3′	5′-GAGCGGATGAAGGTAAAGCG-3′
Il4ra	5′-CACCTGGAGTGAGTGGAGTC-3′	5′-AGGCAAAACAACGGGATG-3′
Il6	5′-CCAGAGATACAAAGAAATGATGG-3′	5′- ACTCCAGAAGACCAGAGGAAAT-3′
Irf4	5′-AATGGGAAACTCCGACAGTG-3′	5′-TAGGAGGATCTGGCTTGTCG-3′
Irf5	5′-AATACCCCACCACCTTTTGA-3′	5′-TTGAGATCCGGGTTTGAGAT-3′
Nos2	5′-GTTCTCAGCCCAACAATACAAGA-3′	5′-GTGGACGGGTCGATGTCAC-3′
Ntf5	5′-CAGCCGGGGAGCAGAGAA-3′	5′- ACACCTGTCAACAGCAGCAC-3′
Pparg	5′-CCATTCTGGCCCACCAAC-3′	5′-AATGCGAGTGGTCTTCCATCA-3′
Socs3	5′-GCTCCAAAAGCGAGTACCAGC-3′	5′-AGTAGAATCCGCTCTCCTGCAG-3′
Stat1	5′-TGGTGAAATTGCAAGAGCTG-3′	5′-CAGACTTCCGTTGGTGGATT-3′
Stat3	5′-GCACCTTGGATTGAGAGTCA-3′	5′-CCCAAGAGATTATGAAACACCA-3′
Stat6	5′-TCTCCACGAGCTTCACATTG-3′	5′-GACCACCAAGGGCAGAGAC-3′
Tgfb1	5′-GTCAGACATTCGGGAAGCAG-3′	5′-GCGTATCAGTGGGGGTCA-3′
Tnfa	5′-CTGTGAAGGGAATGGGTGTT-3′	5′-GGTCACTGTCCCAGCATCTT-3′
Trem2	5′-TTGCTGGAACCGTCACCATC-3′	5′-CACTTGGGCACCCTCGAAAC-3′
